# Induction of Cellular Senescence by Doxorubicin Is Associated with Upregulated *miR-375* and Induction of Autophagy in K562 Cells

**DOI:** 10.1371/journal.pone.0037205

**Published:** 2012-05-11

**Authors:** Ming-Yu Yang, Pai-Mei Lin, Yi-Chang Liu, Hui-Hua Hsiao, Wen-Chi Yang, Jui-Feng Hsu, Cheng-Ming Hsu, Sheng-Fung Lin

**Affiliations:** 1 Graduate Institute of Clinical Medical Sciences, College of Medicine, Chang Gung University, Tao-Yuan, Taiwan; 2 Division of Hematology-Oncology, Department of Internal Medicine, Kaohsiung Medical University Hospital, Kaohsiung, Taiwan; 3 Department of Nursing, I-Shou University, Kaohsiung, Taiwan; 4 Faculty of Medicine, Kaohsiung Medical University, Kaohsiung, Taiwan; 5 Department of Otolaryngology, Kaohsiung Chang Gung Memorial Hospital and Chang Gung University College of Medicine, Kaohsiung, Taiwan; National Taiwan University, Taiwan

## Abstract

**Background:**

Cellular senescence is a specialized form of growth arrest that is generally irreversible. Upregulated *p16*, *p53*, and *p21* expression and silencing of E2F target genes have been characterized to promote the establishment of senescence. It can be further aided by the transcriptional repression of proliferation-associated genes by the action of HP1γ, HMGA, and DNMT proteins to produce a repressive chromatin environment. Therefore, senescence has been suggested to functions as a natural brake for tumor development and plays a critical role in tumor suppression and aging.

**Methodology/Principal Findings:**

An *in vitro* senescence model has been established by using K562 cells treated with 50 nM doxorubicin (DOX). Since *p53* and *p16* are homozygously deleted in the K562 cells, the DOX-induced senescence in K562 cells ought to be independent of *p53* and *p16-pRb* pathways. Indeed, no change in the expression of the typical senescence-associated premalignant cell markers in the DOX-induced senescent K562 cells was found. MicroRNA profiling revealed upregulated *miR-375* in DOX-induced senescent K562 cells. Treatment with *miR-375* inhibitor was able to reverse the proliferation ability suppressed by DOX (*p*<0.05) and overexpression of *miR-375* suppressed the normal proliferation of K562 cells. Upregulated *miR-375* expression was associated with downregulated expression of *14-3-3zeta* and *SP1* genes. Autophagy was also investigated since DOX treatment was able to induce cells entering senescence and eventually lead to cell death. Among the 24 human autophagy-related genes examined, a 12-fold increase of *ATG9B* at day 4 and a 20-fold increase of *ATG18* at day 2 after DOX treatment were noted.

**Conclusions/Significance:**

This study has demonstrated that in the absence of *p53* and *p16*, the induction of senescence by DOX was associated with upregulation of *miR-375* and autophagy initiation. The anti-proliferative function of *miR-375* is possibly exerted, at least in part, by targeting *14-3-3zeta* and *SP1* genes.

## Introduction

Cellular senescence is a specialized form of terminal differentiation that it is generally irreversible and is associated with characteristic alterations in morphology, physiology, gene expression [1]–[4], a typical upregulated senescence-associated-β-galactosidase (SA-β-gal) activity [5], and novel changes in chromatin architecture, i.e. the formation of senescence-associated heterochromatic foci (SAHF) [6]. It is believed that cellular senescence played a role in tumor suppression and aging [6] since the accumulation of senescent cells, the disturbance of the microenvironment, and the resulted compromised tissue function were often observed in age-related pathologies [6], [7]. Recent studies have identified *Rb*, *p53*, and *Skp2* as critical genes common to initiation, execution and maintenance of senescence-associated growth arrest [8], [9]. However, the mechanisms responsible for the alterations of gene expression during cellular senescence remained unclear.

MicroRNAs (miRNAs) are short (19 to 23 nucleotides) non-coding RNAs that are cleaved from 70- to 100-nucleotide hairpin-shaped precursors and act to decrease protein synthesis through translational repression or mRNA degradation [10], [11]. Therefore, miRNAs are crucial factors of diverse regulation pathways, including development, cell differentiation, proliferation and apoptosis [12]–[15] and miss-regulation of miRNA expression contributes to many human diseases and cancers [16]–[19]. MiRNAs have also been implicated in cellular senescence and organismal aging since changes in miRNA expression levels and their putative targets were observed [20]–[24].

Chronic myeloid leukemia (CML) was characterized by Philadelphia (Ph) chromosome that generates a unique *BCR-ABL* fusion gene. In the p210 *BCR-ABL* fusion gene, the down-regulated tyrosine kinase located on the ABL protein, was constitutively activated by the fused BCR gene. The activated tyrosine kinase then signals various pathways, resulting in increased cell proliferation and resistance to apoptosis induced by chemotherapeutics. K562 cell line was a well-characterized model system for human p210 *BCR-ABL*-positive CML with homozygously deleted *p53* and *p16* genes [25], [26]. Doxrubicin (DOX) was commonly used in combined therapy for treating leukemias, Hodgkins’s lymphoma, multiple myeloma, and other solid tumors [27] but not for blastic crisis-phase CML because it fails to induce apoptosis of CML cells [28]. In this study, the molecular mechanism of DOX-induced cellular senescence in K562 cells was investigated. The *in vitro* senescence model was established by using K562 cells treated with DOX. In the absence of *p53* and *p16-pRb*, the induction of cellular senescence by DOX in K562 cells was found to be associated with upregulation of *miR-375*, downregulation of *14-3-3zeta* and *SP1* genes, and the initiation of autophagy.

## Results

### DOX Induced Senescence in K562 Cells

**Figure 1:**
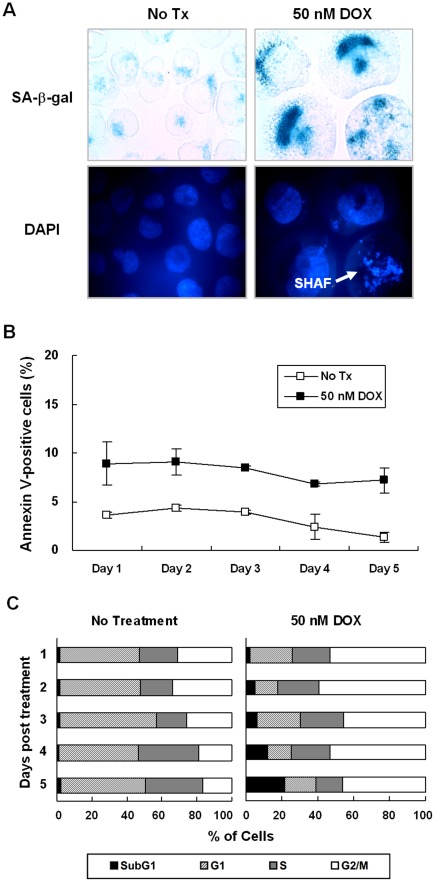
DOX induced senescence but PTX not senescence in K562 cells. (A) K562 cells treated with 50 nM of DOX for 4 days were stained for SA-β-gal activity followed by DAPI staining. Original magnification is 400×. Representative microscopic fields are shown. (B) K562 cells were treated with 50 nM of DOX for 5 days, and the percentages of apoptotic cells were determined by Annexin V/PI staining followed by flow cytometric analysis. Data represented are the means and SE of 3 independent experiments. (C) K562 cells were treated with 50 nM of DOX for 5 days, and DNA contents were measured by flow cytometric analysis after PI staining. Data represented are the means and SE of 3 independent experiments.

To establish an *in vitro* cellular senescence model, K562 cells were treated with 50 nM of DOX. The alterations in cell morphology [1], upregulated SA-β-gal activity^5^ and SAHF formation [29] were used as markers to evaluate cellular senescence. A significantly enlarged cell size, increased SA-β-gal activity, and increased SAHF in cells treated with 50 nM DOX for 4 days were noted (Figure 1A). Percentage of Annexin V-positive cells remained low in K562 cells treated with 50 nM DOX (Figure 1B). Cell cycle analysis revealed that 50 nM DOX caused K562 cells to accumulated in G_2_/M phase (Figure 1C). By treating K562 cells with 50 nM DOX for 4 and 5 days, we have established an *in vitro* senescence model system.

### Expression of Senescence-associated Genes did not Change in DOX-induced Senescent K562 Cells

**Figure 2:**
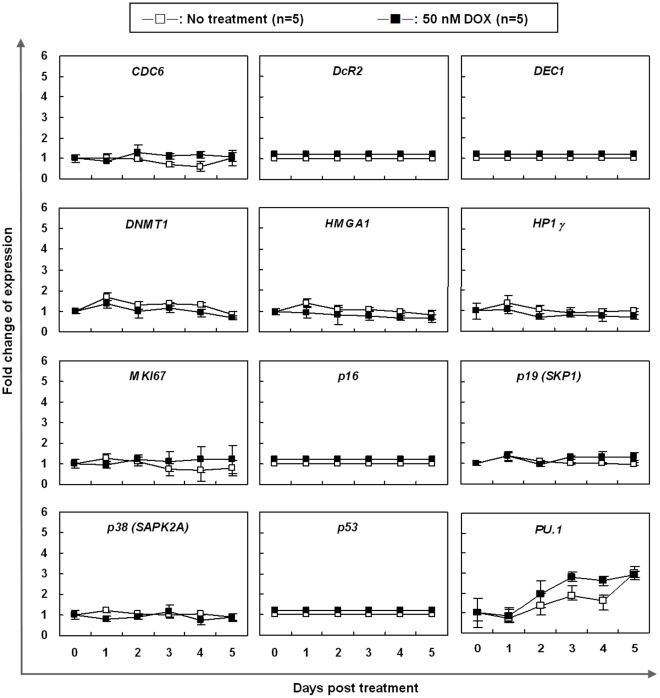
mRNA expression of senescence-associated genes in K562 cells treated with 50 nM DOX as measured by real-time quantitative RT-PCR. The x-axis indicates the days post DOX treatment and the y-axis represents the relative mRNA expression level. The value of the mRNA expression at day 0 is designated 1, and the levels of all other days are calibrated to this value. Data represented are the means and SE of 5 independent experiments.

Expression of *p16^INK4a^* and *p14^ARF^* [30], [31], and excess activity of p53 [32] have been suggested to be biomarkers for aging. In addition, some other senescence-associated genes such as *CDC6,* its overexpression was reported to be sufficient to induce DNA damage and senescence [33]. In some cells, senescence is associated with global changes in chromatin structure which leads to the accumulation of heterochromatin protein 1 (HP1), histone H3 trimethylated on lysine 9 (me-K9H3) in SAHF, and on the promoters of certain cell-cycle genes [29], [34]. The decision to enter cellular senescence was determined by a histone methyltransferase (HMT) that acts with Rb and HP1 proteins to alter chromatin structure and silencing E2F target genes. HMGA proteins cooperate with the p16^INK4a^ tumor suppressor to promote SAHF formation, proliferation arrest, and senescence commitment by contributing to the repression of proliferation-associated genes [35]. Therefore, further investigation on the changes of these “classical" senescence molecular markers (Table S1) in DOX-induced senescent K562 cells is needed. Since *p53* and *p16* were homozygously deleted in the K562 cells, the expression of *p53* and *p16* was not detected in 50 nM DOX-treated K562 cells as expected (Figure 2). Unexpectedly, the mRNA expression of senescence-associated genes, *CDC6*, *DcR2*, *DEC1*, *DNMT1*, *HMGA1*, *HP1γ*, *MKi67*, *p19*, *p38*, *p53*, and *PU.1*, remained unchanged between the untreated and 50 nM DOX-treated K562 cells for up to 5 days (Figure 2).

### Identification of miRNAs Differentially Expressed in DOX-induced Senescent K562 Cells

**Figure 3:**
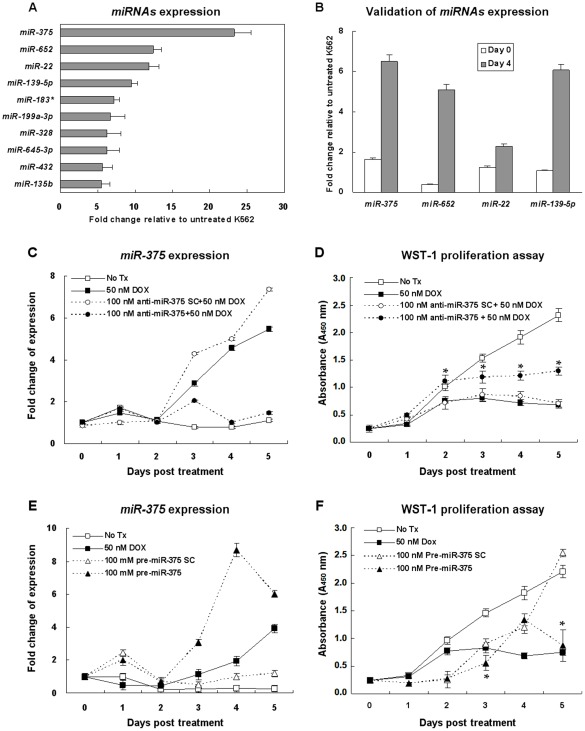
*miR-375* is upregulated in DOX-induced senescent K562 cells. (A) miRNAs upregulated in 50 nM DOX-treated K562 cells for 4 days as measured by TaqMan® microRNA microarray analysis. The value of the miRNA expression in untreated K562 cells of day 4 is designated 1, and the level of miRNA expression of DOX-treated K562 cells are calibrated to this value. Data represented are the means and SE of 3 independent experiments. (B) Validation of miRNA expression by individual mature TaqMan® microRNA assays using real-time quantitative RT-PCR. The 4 most strongly expressed miRNAs selected from TaqMan® microRNA microarray analysis were further validated. The value of the miRNA expression in untreated K562 cells is designated 1, and the level of miRNA expression of DOX-treated K562 cells of the same day are calibrated to this value. Data represented are the means and SE of 3 independent experiments. (C) Inhibition of *has*-*miR-375* by 100 nM *has*-anti-*miR-375* inhibitor or 100 nM *has*-anti-*miR-375* inhibitor scramble negative control (SC) in K562 cells. After transfection for 48 hours, K562 cells were treated with 50 nM DOX for 5 days. The expression of mature *has*-*miR-375* was examined by TaqMan® microRNA assays using real-time quantitative RT-PCR. The value of the *has*-*miR-375* expression at day 0 is designated 1, and the levels of all other days of the same treatment are calibrated to this value. Data represented are the means and SE of 5 independent experiments. (D) WST-1 assay was performed to determine cell proliferation after 100 nM *has-*anti-*miR-375* inhibitor or 100 nM *has*-anti-*miR-375* inhibitor SC transfection followed by 50 nM DOX treatment in K562 cells. Data represented are the means and SE of 5 independent experiments. *Indicates significant difference compared to cells treated with 50 nM DOX and treated with 100 nM anti*-miR-375* SC and 50 nM DOX (*p*<0.05). (E) Overexpression of *miR-375* by 100 nM *has*-*miR-375* precursor or 100 nM *has*-*miR-375* precursor SC in K562 cells. The measurement and calculation of mature *has*-*miR-375* expression were as described in (C). Data represented are the means and SE of 5 independent experiments. (F) WST-1 assay was performed to determine cell proliferation after 100 nM *has*-*miR-375* precursor or 100 nM *has*-*miR-375* precursor SC transfection in K562 cells. Data represented are the means and SE of 5 independent experiments. *Indicates significant difference compared to both untreated K562 cells and cells treated with 100 nM *has*-*miR-375* precursor SC (*p*<0.05).

To further elucidate the regulatory mechanisms of DOX-induced senescence, TaqMan® microRNA microarray system was used to cover a total of 667 human miRNAs, for the analysis of miRNA expression profiles of K562 cells treated or not treated with 50 nM DOX for 4 days from three independent experiments. By comparing miRNA expression profiles between treated and untreated K562 cells, 10 up-regulated miRNAs were found (at least four-fold increase) in DOX-treated K562 cells (Figure 3A). Four most strongly expressed miRNAs, *miR-375*, *miR-652*, *miR-22*, and *miR139-5p,* were selected for further validation by using individual TaqMan® microRNA assays. The expression of *miR-375* remained to be the highest among the 4 miRNAs (Figure 3B). *miR-375* was chosen for further study due to its consistently high overall expression in DOX-treated K562 cells.

### Inhibition of *miR-375* can Partially Reverse the Proliferation Ability Suppressed by DOX in K562 Cells

To explore the function of *miR-375* in DOX-induced senescence, K562 cells were transfected with *has*-anti-*miR-375* inhibitor or *has*-anti-*miR-375* inhibitor scramble negative control followed by 50 nM DOX treatment for 5 days. The expression of *miR-375* after transfection was checked to confirm the transient knockdown of *miR-375* by *has*-anti-*miR-375* inhibitor (Figure 3C). As shown in Figure 3D, in cells transfected with *has*-anti-*miR-375* inhibitor, cell proliferation was partially restored when compared with untreated cells. It was significantly higher in cells transfected with *has*-anti-*miR-375* inhibitor scramble negative control as compared to cells treated with DOX only (*p*<0.05). K562 cells were also transfected with *has*-*miR-375* precursor or *has*-*miR-375* precursor scramble negative control to investigate the function of *miR-375* in cellular senescence. The expression of mature *miR-375* was increased at post *has*-*miR-375* precursor transfection day 3 and persisted up to day 5. A decreased in cell proliferation followed by an increased in mature *miR-375* expression was observed in *has*-*miR-375* precursor-treated K562 cells (Figure 3F).

### Downregulation of Putative *miR-375* Target Genes, *14-3-3zeta* and *SP1*, was Associated with *miR-375* Upregulation in DOX-induced Senescent K562 Cells

**Figure 4:**
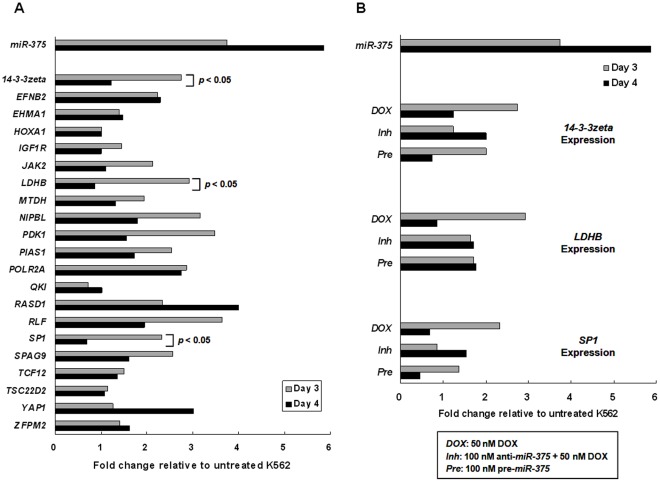
Expression of putative *miR-375* target genes in DOX-induced senescent K562 cells. (A) mRNA expression of putative *miR-375* target genes in K562 cells treated with 50 nM DOX for 3 and 4 days as measured by real-time quantitative RT-PCR. The value of the mRNA expression in untreated K562 cells of the same day is designated 1, and the level of mRNA expression of DOX-treated K562 cells are calibrated to this value. Data represented are the means and SE of 5 independent experiments. (B) Expression of *14-3-3zeta*, *LDHB*, and *SP1* genes in K562 cells treated with 50 nM DOX (*DOX*) transfected with 100 nM *has-*anti-*miR-375* inhibitor followed by 50 nM DOX treatment (*Inh*) or transfected with 100 nM *has*-*miR-375* precursor (*Pre*) for 3 and 4 days. The calculation of gene expression was as described in (A). Data represented are the means and SE of 3 independent experiments.

To further identify the targets of *miR-375*, published literatures were searched and 21 putative *miR-375* target genes were found by using TargetScan, PicTar and miRanda algorithms (Table S2). The expression of these 21 genes in K562 cells treated with 50 nM DOX for 3 and 4 days were analyzed, and the expression levels of *14-3-3zeta*, *LDHB*, and *SP1* genes were found to be diminished (*p*<0.05) as *miR-375* increased (Figure 4A). In cells transfected with *has*-anti-*miR-375* inhibitor followed by 50 nM DOX treatment or transfected with *has*-*miR-375* precursor, the expression of *14-3-3zeta* and *SP1* genes was inversely associated with the down- or up-regulated expression of *miR-375* (Fig. 4B). In contrast, the expression of *LDHB* was not affected by the levels of *miR-375* (Fig. 4B). These results suggested that *14-3-3zeta* and *SP1* genes are the possible targets of *miR-375* in DOX-treated senescent K562 cells.

### Upregulation of *miR-375* was Associated with Upregulated *ATG9B* and *ATG18* in DOX-induced Senescent K562 Cells

**Figure 5:**
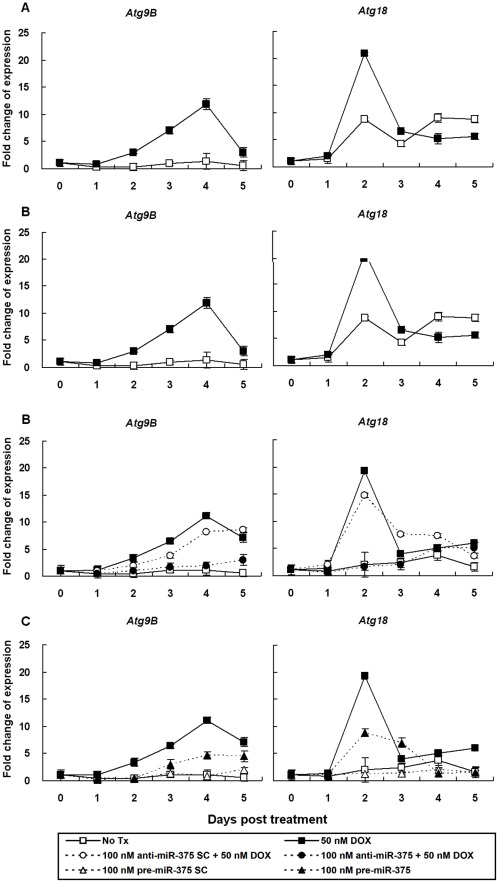
Upregulated *ATG9B* and *ATG18* in DOX-treated K562 cells as measured by real-time quantitative RT-PCR. (A) Expression of *Atg9B* and *Atg18* in K562 cells treated or not treated with 50 nM DOX for 5 days. (B) Expression of *Atg9B* and *Atg18* in K562 cells transfeced with 100 nM *has*-anti-*miR-375* inhibitor or 100 nM *has*-anti-*miR-375* SC followed by 50 nM DOX for 5 days. (C) Expression of *Atg9B* and *Atg18* in K562 cells transfeced with 100 nM *has*-*miR-375* precursor or 100 nM *has*-*miR-375* precursor SC for 5 days. The x-axis indicates the days post DOX treatment and the y-axis represents the relative mRNA expression level. The value of the mRNA expression at day 0 is designated 1, and the levels of all other days are calibrated to this value. Data represented are the means and SE of 5 independent experiments.

With the observation that DOX treatment inducing cells senescence and the eventual cell death, the alternative mode of cell death, autophagy, was also investigated. The expression of 24 autophagy-related genes (Table S3 and Figure S1) using real-time quantitative RT-PCR were analyzed. A 12-fold increase of *ATG9B* at day 4 and a 20-fold increase of *ATG18* at day 2 was observed in DOX-treated K562 cells (Figure 5 A and B). Cells transfected with *has*-anti-*miR-375* inhibitor followed by 50 nM DOX treatment for 5 days did not showed the fluctuated expression of *ATG9B* and *ATG18* (Figure 5 C and D). Overexpression of *miR-375* by *has*-*miR-375* precursor transfection resulted in an elevated expression of *ATG9B* and of *ATG18* with a similar patterns as observed in DOX-treated K562 cells (Figure 5 E and F). Our results suggested that upregulation of *miR-375* were associated with the induction of autophagy in the DOX-induced senescence.

## Discussion

The understanding of cellular responses induced by chemotherapeutic drugs provides useful insights in designing regimens for cancer treatment. In this study, K562 cells were used as a model of advanced CML to examine the cellular responses induced by DOX and further investigated the mechanisms of DOX-induced senescence. Due to the lack of both *p16* and *p53* genes, K562 cells can also serve as a model for examining the *p16-* and *p53-*independent pathways activated by chemotherapeutic drugs.

In this study, an *in vitro* senescence model using DOX to treat K562 cells were to be established. Based on previous report [36], we have also found that senescence was induced at 50 nM DOX, but not apoptosis. DOX is a chemotherapeutic drugwith a wide range of cellular targets [27] and can stimulate differentiation [37] of K562 cells. It has been suggested that differentiation induced by DOX is caspases-dependent [36], but the mechanism remains elusive.

In addition to differentiation, DOX was also able to induce senescence in various cancers cells, such as CML [36] and breast cancer [38]. The characteristics of cellular senescence, including increased expression of SA-β-gal, cell enlargement, and SHAF formation, were also observed in our study. Changes in gene expression, such as upregulated *p16*, *p53*, and *p21* expression and silencing of E2F target genes, have been characterized to promote the establishment of senescence [29]. It can be further aided by the transcriptional repression of proliferation-associated genes by the action of HP1γ [29], [34], HMGA [35], and DNMT [35] proteins to produce a repressive chromatin environment. In addition, the DOX-induced senescence in K562 cells should be independent of *p53* and *p16-pRb* pathways, since *p53* and *p16* are homozygously deleted in the K562 cells. Indeed, the expression of the typical SA-premalignant cell markers (*CDC6*, *DEC1*, *DcR2*, *DNMT1*, *HMGA1*, *HP1γ, Ki67*, *p19*, *p38*, and *PU1*) remained unchanged in the DOX-induced senescent K562 cells.

An increase of *miR-375* expression in DOX-induced senescent K562 cells was also observed. Our study has coincided with an overall low level of miRNA population in untreated K562 cells as described by a recent study [39]. In our DOX-induced senescent K562 cells, treatment with *miR-375* inhibitor could partially rescue the cellular proliferation suppressed by DOX. Over-expression of *miR-375* was shown to suppress the normal proliferation of K562 cells. A recent study has also demonstrated that *miR-375-*down-regulated gastric carcinoma cell line treated with both 5-aza-2′-deoxycytidine and Trichostatin A could upregulate *miR-375* expression and reduced the cell viability [40]. In fact, down-regulated *miR-375* has been reported in various types of cancers, including prostate [41], oral and pharyngeal [42], head and neck [43], gastric [40], and hepatocellular [44] carcinomas. However, its function in these cancers and the mechanism responsible for its down-regulation remained unknown. Based on these results, *miR-375* could play a protective role in tumorigenesis and possibly through the induction of cell senescence.

Recent studies have identified targets of *miR-375* in various types of cancers, such as Yes-associated protein (YAP) in liver cancer [45], *MTDH/AEG-1* in head and neck squamous cell carcinoma and hepatocellular carcinoma [46], [47], *IGF1R* and *PDK1* in esophageal squamous cell carcinoma [48], [49], *LDHB* in maxillary sinus squamous cell carcinoma [50], *JAK2*, *PDK1*, and *14-3-3zeta* in gastric cancer [40], [51], [52], and *SP1* in cervical cancer [53]. In this present study, we observed an association between upregulated *miR-375* and downregulated *14-3-3zeta* and *SP1* genes. *14-3-3zeta* is a potent anti-apoptotic gene and *SP1* is a transcriptional regulator. Both *14-3-3zeta* and *SP1* genes have been shown to participate in cancer development and progression [40], [53]. It is therefore reasonable to hypothesize that downregulation of *miR-375* results in enhanced expression of *14-3-3zeta* and *SP1* and provides a survival advantage for cancer cells, in contrast, upregulation of *miR-375* diminishes the expression of *14-3-3zeta* and *SP1* and leads to cellular senescence. Both *miR-375* and its target genes, *14-3-3zeta* and *SP1,* might be therapeutic targets, and either restoring *miR-375* expression or abolishing expression of *14-3-3zeta* and *SP1* genes could diminish malignant cell behaviors and consequently block the progression of cancer. In addition, identification of *miR-375* targets should help us to further elucidate the alternative pathway that is responsible for the DOX-induced senescence in the absence of both *p16* and *p53* genes.

Cellular senescence and autophagy are two different cellular responses to stress. Autophagy is a genetically programmed process of non-apoptotic cell death that degrades long-lived cellular proteins and organelles. Recent study has shown that autophagy is activated during the process of senescence and a subset of autophagy-related genes is upregulated during senescence [54]. In this study, DOX has induced senescence in K562 cells but the cells eventually died. It is therefore logical to hypothesize that autophagy was involved in the process of non-apoptotic cell death after cellular senescence. Indeed, a 12-fold increase of *ATG9B* at day 4 and a 20-fold increase of *ATG18* at day 2 after DOX treatment were observed. *ATG9* is the only integral membrane component of the conserved ATG machinery and was suggested to aid in the search for the source of the pre-autophagosomal structure [55]. *ATG18* is a phosphatidylinositol 3-phosphate-binding protein and is required for both the cytoplasm to vacuole targeting (Cvt) pathway and autophagy [56]. In autophagy, *ATG18* is recruited early to form autophagosome. Hence, upregulated *ATG9B* and *ATG18* implies the initiation of autophagy in DOX-induced cellular senescence in K562 cells which is consistent with the finding that autophagy is activated during the process of senescence [54]. Autophagy has been shown to suppress tumor progression by limiting chromosomal instability [57]. From the view of tumor suppression, both cellular senescence and autophagy may act cooperatively to exert their functions as natural brake to tumor development.

In summary, cellular senescence induced by DOX is associated with upregulated *miR-375* expression and autophagy initiation in the absence of *p16* and *p53* genes. The anti-proliferative function of *miR-375* is possibly exerted, at least in part, by targeting *14-3-3zeta* and *SP1* genes. This study provides extended understanding for the molecular mechanisms of *p16-* and *p53*-independent cellular senescence. Further study on the cellular senescence pathways regulated by *miR-375* and the mechanism of autophagy initiated by DOX should provide insights for better cancer therapy.

## Materials and Methods

### Cell Line and Drug Treatment

Chronic myeloid leukemic cell line K-562 was purchased from Food Industry Research and Development Institute, Taiwan. Cells were maintained in RPMI 1640 medium (Invitrogen) supplemented 10% HyClone fetal bovine serum (Thermo Scientific) and grown at 37°C with 5% CO_2_. Stock solutions (1 mM) of DOX (D-1515, Sigma-Aldrich) was stored in the dark at −20°C and diluted in RPMI 1640 medium immediately before treating cells.

### β-galactosidase (β-gal) and DAPI Staining

For cytospin preparation, 5×10^5^ cells were washed in PBS and cytocentrifuged (350 rpm, 5 min) onto glass slides, then fixed in 0.5% glutaraldehyde/PBS for 5 min. After fixation, cells were washed twice by phosphate-buffered saline (PBS) and incubated in fresh senescence-associated β-Gal (SA-β-gal) staining solution [5-bromo-4chloro-3-indolyl β-D-galactoside (X-Gal) 1 mg/mL, K_3_Fe[CN]_6_ 0.21 mg/mL, K_4_Fe[CN]_6_ 0.16 mg/mL, MgCl_2_ 2 mM] at 37°C without CO_2_ for 24 hr. After SA-β-Gal staining, cells were washed twice with PBS, twice with H_2_O, and stained with DAPI (10 µL/mL) for 10 min for DNA visualization.

### Cell Proliferation Assays

Cell proliferation was evaluated using Premixed WST-1 Cell Proliferation Reagent (Clontech) based on the cleavage of tetrazolium salt WST-1 (4-[3-(4iodophenyl)-2- (4-nitrophenyl)-2H-5-tetrazolio]-1,3- benzene disulfonate) into formazan by cellular mitochondrial mitochondrial succinate-tetrazolium reductase in viable cells. Briefly, 100 µL of cells of different treatments were plated in triplicates in a 96-well plate and 10 µL WST-1 Cell Proliferation Reagent was added to each well. Cells were incubated in a humidified atmosphere at 37°C in 5% CO_2_ for 30 min, the 96-well plate was shaken thoroughly for 1 min, and absorbance was read at 450 nm using a microplate reader. The background absorbance was measured in wells containing only the dye solution and culture medium. Data presented were the absorbance values subtracted by the background absorbance values and the mean of the triplicates were calculated.

### Flow Cytometry

Flow cytometric analysis of stained cells was performed on a FACSCalibur flow cytometer (Becton Dickinson). Percentages of apoptotic cells were assessed by dual staining of cells with Annexin V and propidium iodide (PI). Cells (1×10^5^) were washed in cold PBS and resuspended in 200 µL staining solution containing 5 µL of Annexin V-fluorescein isothiocyanate (FITC) and 10 µL of 20 µg/mL PI (BD Pharmingen). Cell cycle analysis was performed on PI-stained cells and the percentages of the cell population in subG_1_, G_1_, S or G_2_/M phases were calculated from histograms using WinMDI 2.9 software.

### MicroRNA Microarray Analysis

K562 cells treated with or without 50 nM DOX (Sigma-Aldrich) for four days were used for microRNA microarray analysis. Total RNAs were extracted using TriZol (Invitrogen) and reverse transcription (RT) was performed using the TaqMan® MicroRNA Reverse Transcription Kit (Applied Biosystems) in a final volume of 7.5 µL containing 1 µg of RNA, 1× Megaplex™ RT primers human pool A or B (Applied Biosystems), 2.5 mM dNTPs with dTTP, 0.01 U MultiScribe Reverse Transcriptase, 1× Reverse Transcription Buffer, 3 mM MgCl_2_, and 0.25 U RNase inhibitor. The RT products were then subjected for miRNA expression profiling using TaqMan® Human MicroRNA array A and B (PN 4398977; Applied Biosystems) on an Applied Biosystems 7900HT Sequence Detection System (Applied Biosystems). PCR cycling parameters were set as follows: 95°C for 10 min followed by 50 cycles of PCR reactions at 95°C for 10 sec, 60°C for 40 sec, and 72°C for 1 sec. The expression levels of the 667 human mature miRNAs were normalized to *U6 snRNA* internal control and relative expression levels were calculated by the comparative Ct (ΔΔCt) method.

### MicroRNA Expression Analysis

The mature microRNA expression was quantified in real-time quantitative RT-PCR systems using TaqMan® microRNA assays according to the manufacturer’s protocols (Applied Biosystems). Briefly, RT reactions were performed with 10 ng of total RNA, 50 nM stem-loop microRNA- specific RT primers, 1× RT buffer, 0.25 mM of dNTPs, 3.33 U/µL MultiScribe RTase and 0.25 U/µL RNase inhibitor. The reaction mixture was incubated for 30 min at 16°C and 30 min at 42°C, followed by 5 min incubation at 85°C to inactivate the RTase enzyme. RT products were subjected to microRNA expression assay for real-time quantitative PCR in a 20-µL final volume containing 2 µL of RT product, 1 µL of 20× TaqMan® microRNA Assay (Applied Biosystems), and 10 µL of 2× TaqMan® Universal PCR Master Mix (Applied Biosystems). The PCR cycling parameters were 95°C for 15 sec followed by 60°C for 30 sec for 40 cycles. *U6 snRNA* TaqMan® miRNA assay (Applied Biosystems) was used as endogenous control for microRNA expression analysis. Real-time quantitative PCR was performed in a 7500 Fast Real-Time System (Applied Biosystems) and the relative gene expression levels were calculated by the comparative Ct (ΔΔCt) method.

### Transient Transfections

Transfection experiments of K562 cells with anti-miR™ *has-miR-375* inhibitor (Ambion), anti-miR™ miRNA inhibitors negative scramble control (Ambion), 100 nM *has*-*miR-375* precursor (Ambion), and 100 nM *has*-*miR-375* precursor negative scramble control (Ambion) were carried out using siPORT NeoFX Transfection Agent (Ambion). Briefly, 10^6^ cells were plated in 10-cm culture dishes and different amounts of RNAs and siPORT NeoFX Transfection Agent diluted in OPTI-MEM® I medium (Invitrogen) were added to cells and incubated at 37°C with 5% CO_2_. Cells were harvested 48 h after transfection, counted, and plated 10^4^ cells/well in 6-well plates for further drug treatment experiments.

### Real-time Quantitative RT-PCR Analysis

RNA samples were extracted using TriZol reagent (Invitrogen). The 2 µg RNA input for cDNA synthesis was determined by spectrophotometric OD_260_ measurement and cDNA was generated using High Capacity cDNA Reverse Transcription Kit (Applied Biosystems) according to the manufacture’s protocols. The expression of senescence associated genes and putative *miR-375* target genes were analyzed using TaqMan® system. The gene names, GenBank accession numbers, and assay ID of gene expression assays or primer sequences of senescence-associated genes and putative *miR-375* target genes are list in Table S1 and S2, respectively. Expression of human housekeeping genes, *ACTB* (β-actin), *GAPDH* (glyceraldehyde- 3-phosphate dehydrogenase), *HPRT* (hypoxanthine phosphoribosyltransferase), *18S* (18S ribosomal RNA), *TBP* (TATA box binding protein) and *POLR2A* (RNA polymerase II polypeptide A) were evaluated and validated for normalizing RNA expression in real-time quantitative RT-PCR of senescence-associated genes and *miR-375* target genes (Figure S2). All 6 TaqMan® endogenous controls were purchased from Applied Biosystems. Reactions were carried out in a 20-µL final volume containing 50 ng cDNA (as total input RNA), 1 µL 20× TaqMan® Gene Expression Assay, and 10 µL 2× TaqMan® Universal PCR Master Mix (Applied Biosystems). The expression of autophagy-related genes (ATG) was analyzed using SYBR® Green system. The gene names, GenBank accession numbers, amplicon sizes, and sequences of forward and reverse primers are listed in Table S3. Reactions were carried out in a 20-µL final volume containing 50 ng cDNA (as total input RNA), 200 nM each primer, and 10 µL 2× *Power* SYBR® Green PCR Master Mix (Applied Biosystems). Real-time quantitative PCR was performed in a 7500 Fast Real-Time System (Applied Biosystems) and the PCR cycling parameters were set as follows: 95°C for 10 min followed by 40 cycles of PCR reactions at 95°C for 20 sec and 60°C for 1 min. The relative gene expression levels were calculated by the comparative Ct (ΔΔCt) method.

### Statistical Analysis

Results were expressed as mean ± SE (standard error). Comparisons were made with *t*-test using the SPSS for Windows Release 13.0 (SPSS, Chicago, IL). Probability value of <0.05 was regarded as difference with statistical significance.

## Supporting Information

Figure S1 mRNA expression of 6 endogenous control genes in leukemic cell lines measured by real-time quantitative RT-PCR. A, The average Ct with standard deviation (SD). *18S*: 18S ribosomal RNA; *ACTB*: β-actin; *GAPDH*: Glyceraldehyde-3-phosphate dehydrogenase; *HPRT*: Hypoxanthine phosphoribosyl-transferase; *POLR2A*: RNA polymerase II polypeptide A; *TBP*: TATA box binding protein. Error bars are SD. B, Variation of 6 human endogenous controls as measured by SD of Ct. Annotation as for panel A.
(PDF)

Figure S2 mRNA expression of 24 autophagy-related genes in K562 cells treated with 50 nM DOX as measured by real-time quantitative RT-PCR. The value of the mRNA expression at day 0 is designated 1, and the levels of all other days are calibrated to this value. Data represented are the means and SE of 5 independent experiments.
(PDF)

Table S1 TaqMan® Gene Expression Assays for real-time quantitative RT-PCR analysis of the senescence-associated genes.
(PDF)

Table S2 Oligonucleotide primers for real-time quantitative RT-PCR analysis of the putative *miR-375* target genes.
(PDF)

Table S3 Oligonucleotide primers for real-time quantitative RT-PCR analysis of the 24 autophagy-related genes.
(PDF)
