# Supplementary figures and images for: Induction of Cellular Senescence by Doxorubicin Is Associated with Upregulated miR-375 and Induction of Autophagy in K562 Cells
Source: PLoS One. 2012 May 11;7(5):e37205. doi: 10.1371/journal.pone.0037205 (PMC3350486; doi:10.1371/journal.pone.0037205)

(A)

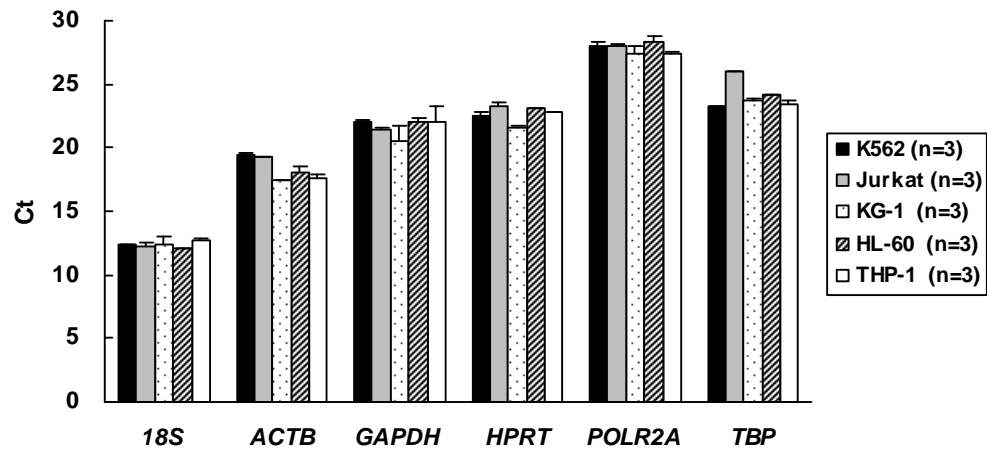

(B)

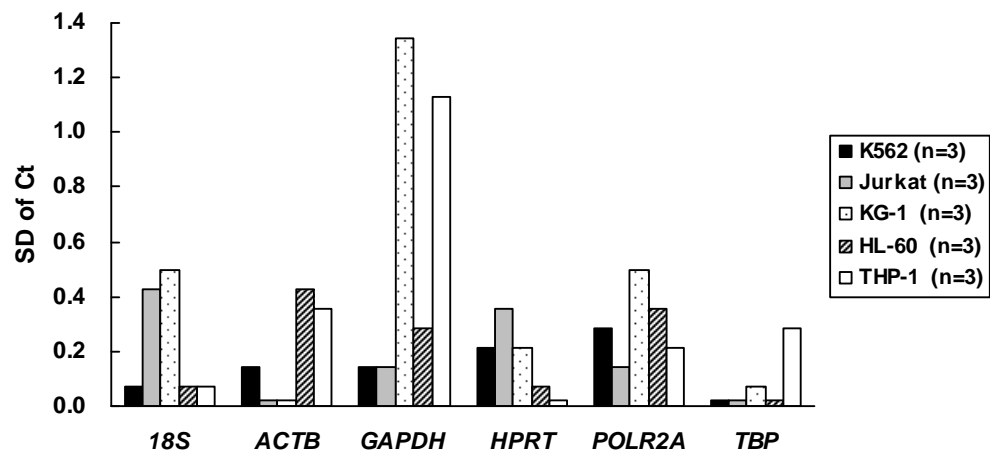

Supplement: Figure S1 — mRNA expression of 6 endogenous control genes in leukemic cell lines measured by real-time quantitative RT-PCR. A, The average Ct with standard deviation (SD). 18S: 18S ribosomal RNA; ACTB: β-actin; GAPDH: Glyceraldehyde-3-phosphate dehydrogenase; HPRT: Hypoxanthine phosphoribosyl-transferase; POLR2A: RNA polymerase II polypeptide A; TBP: TATA box binding protein. Error bars are SD. B, Variation of 6 human endogenous controls as measured by SD of Ct. Annotation as for panel A. (PDF) [file pone.0037205.s001.pdf]

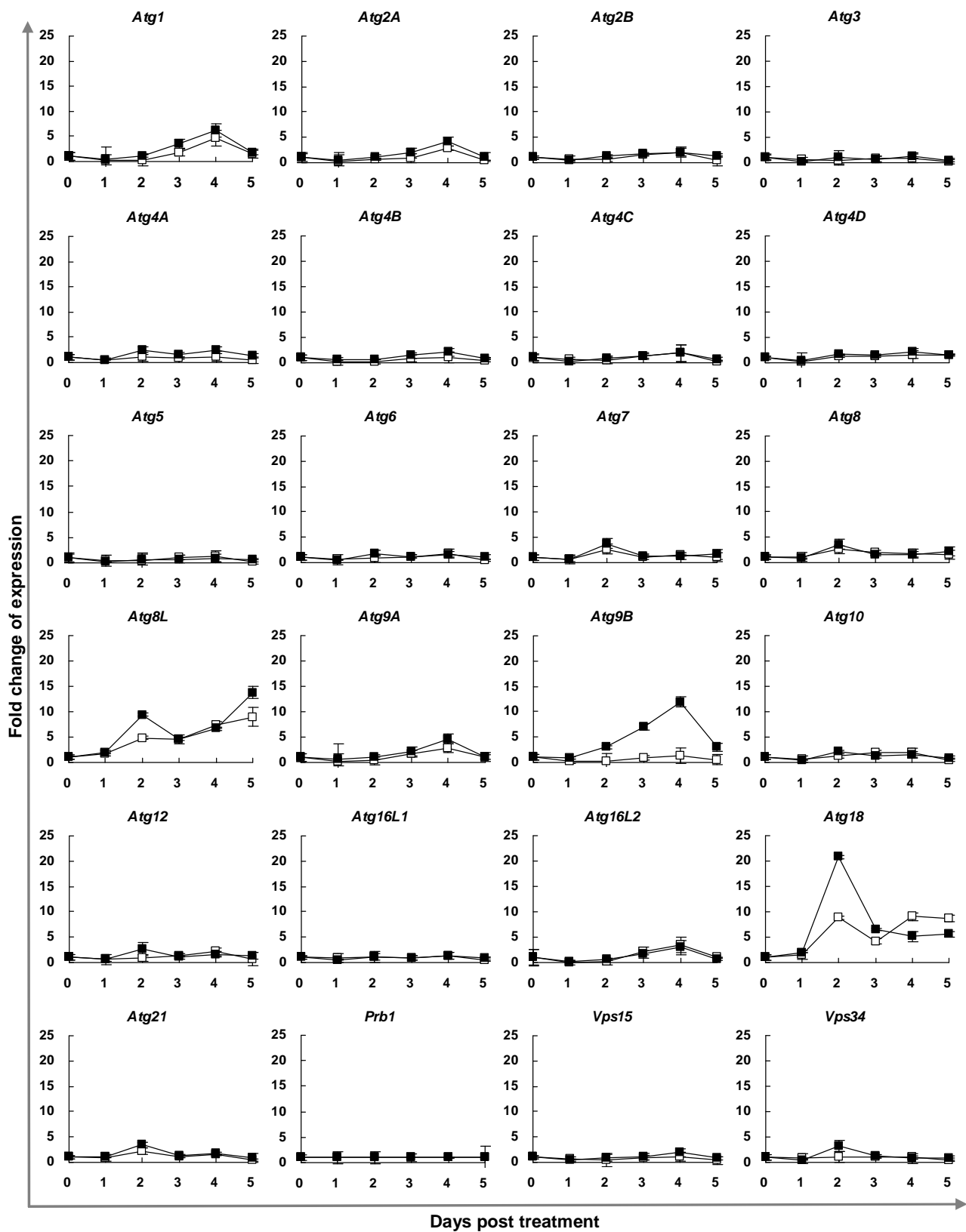

Supplement: Figure S2 — mRNA expression of 24 autophagy-related genes in K562 cells treated with 50 nM DOX as measured by real-time quantitative RT-PCR. The value of the mRNA expression at day 0 is designated 1, and the levels of all other days are calibrated to this value. Data represented are the means and SE of 5 independent experiments. (PDF) [file pone.0037205.s002.pdf]
