# Supplementary material for: Induction of Cellular Senescence by Doxorubicin Is Associated with Upregulated miR-375 and Induction of Autophagy in K562 Cells
Source: PLoS One. 2012 May 11;7(5):e37205. doi: 10.1371/journal.pone.0037205 (PMC3350486; doi:10.1371/journal.pone.0037205)
Supplement: Table S1 — TaqMan® Gene Expression Assays for real-time quantitative RT-PCR analysis of the senescence-associated genes. (PDF) [file pone.0037205.s003.pdf]

Gene	GenBank Accession No.	Amplicon Size (bp)	Assay Location	Assay ID (Applied Biosystems)
CDC6	NM_001254.3	97	1052	Hs01028222_g1
DEC1	NM_017418.2	100	724	Hs00758129_m1
DCR2	NM_031409.3	97	57	Hs01003258_g1
DNMT1	NM_001379.2	98	4904	Hs00945899_m1
HMGA1	NM_145899.2	126	1668	Hs00852949_g1
HMGA2	NM_003483.4	81	1061	Hs00971724_m1
HP1γ	NM_007276.3	130	87	Hs00371848_m1
MKI67	NM_002417.4	96	371	Hs01032438_g1
p15 (CDKN2B)	NM_078487.2	128	642	Hs00365249_m1
p16 (CDKN2A)	NM_058195.3	84	348	Hs00924091_m1
p19A (SKP1)	NM_006930.3	117	176	Hs04186197_g1
p38 (SAPK2A, MAPK14)	NM_139012.2	103	1325	Hs00176248_m1
p53	NM_001126112.1	105	754	Hs01034254_g1
PU.1 (SPI1)	NM_003120.2	103	718	Hs00231368_m1
RIPK1	NM_003804.3	83	1576	Hs00169407_m1
RIPK3	NM_006871.3	70	877	Hs00179132_m1
YAP1	NM_006106.4	64	1069	Hs00371735_m1
