# Supplementary material for: Induction of Cellular Senescence by Doxorubicin Is Associated with Upregulated miR-375 and Induction of Autophagy in K562 Cells
Source: PLoS One. 2012 May 11;7(5):e37205. doi: 10.1371/journal.pone.0037205 (PMC3350486; doi:10.1371/journal.pone.0037205)
Supplement: Table S2 — Oligonucleotide primers for real-time quantitative RT-PCR analysis of the putative miR-375 target genes. (PDF) [file pone.0037205.s004.pdf]

Gene	GenBank Accession No.	Amplicon Size (bp)	Forward primer (5'→3')	Reverse primer (5'→3')	Location
14-3-3zeta	NM_003406	95	AGGTTGCCGCTGGTGATG	TGGTTGCATTTCCTTTTTGCT	530~624
EFNB2	NM_004093	122	GAAGGGACTCCGTGTGGAAGT	AGGTAGAAATTTGGAGTTCGAGGAA	36~157
EHMT1	NM_024757	110	CCAAGAAAGGCCACTACGAAGT	GTACTCTGTGGCCCAGATCATG	2577~2686
HOXA3	NM_153632	80	GCCGCACTAGCGCTTATCTC	GGTTGTTGCTGGCATTCTGA	13~112
IGF1R	NM_000875	95	AGGCTGAATACCGCAAAGTCTT	GGCCACTTGCATGACATCTCT	2194~2288
JAK2	NM_004972	93	ATGTTTGGAGCTTTGGAGTGGTT	TGCCAATCATACGCATAAATTCC	3601~3693
LDHB	NM_001174097	102	GCAGCATGGGAGCTTATTTCTT	CGGACTCCTGCAGTTACCACTA	531~632
MTDH	NM_178812	99	AAGGAGTTGGAGTGACCGTTCA	CGGCTAACATCCCACTGATAATC	1339~1437
NIPBL	NM_133433	111	AGGAGGCACTTCAGGGTCATT	CAGCAAATAGCGATGACATCCA	8533~8643
PDK1	NM_002610	99	ACTATGGAACACCATGCCAACA	TCCTCGGTCACTCATCTTCACA	962~1004
PIAS1	NM_016166	98	GGGCACCGATGAGATCAAAA	CGCTACCTGATGCTCCAATGT	1334~1431
POLR2A	NM_000937	90	CCAACTCTGCACAAAATGTCCAT	CGGAGTTGTCACACTAAGATTCAAG	1770~1859
QKI	NM_006775	80	CCCACAGTTGCAGGATCCAT	AAAACACCGTATAAGCCAAACCA	8138~8217
RASD1	NM_016084	86	GACACGTCCGGCAACCA	TCCAGACTGAACACCAGGATGA	444~529
RLF	NM_012421	85	TCATCTTTTCAGCAAGCCTATATCA	AGTCGACCTTTGCAATCTCCTT	773~857
SP1	NM_003109	110	GGTGGAGAGGAAGGAGAAAACA	GAGCCCCTTCCTTCACTGTCT	1775~1884
SPAG9	NM_001130528	81	CAGCCTGAAGGTCAGCAATAGTC	CCGCCTTGGCTAACATCAGA	941~1021
TCF12	NM_207036	100	CCTAAGCCACCAACCAGTATGTT	GCTGGCTCATCCCATTTGAT	924~1023
TSC22D2	NM_014779	100	GCCTCTGGGATAGTGCATCTG	CTTACTGCATACATCAAATGGCTTTT	2368~2467
ZFPM2	NM_012082	100	TGTAACAAAGGTGATGATGAAGGAA	AGTCGTCTGTCTCAACTCCAGGTT	213~312
