# Supplementary material for: Induction of Cellular Senescence by Doxorubicin Is Associated with Upregulated miR-375 and Induction of Autophagy in K562 Cells
Source: PLoS One. 2012 May 11;7(5):e37205. doi: 10.1371/journal.pone.0037205 (PMC3350486; doi:10.1371/journal.pone.0037205)
Supplement: Table S3 — Oligonucleotide primers for real-time quantitative RT-PCR analysis of the 24 autophagy-related genes. (PDF) [file pone.0037205.s005.pdf]

Gene	GenBank Accession No.	Amplicon Size (bp)	Forward primer (5'→3')	Reverse primer (5'→3')	Location
Atg1	NM_003565	66	TCATCTTCAGCCACGCTGT	CACGGTGCTGGAACATCTC	3204~3269
Atg2A	NM_015104	70	CCAGCAACATCATCATGGAC	CTACTGAGGCGGAACATGGA	3540~3609
Atg2B	NM_018036	61	ACGTTGAAACAGAATGAAGTGC	ACCTACAACTACCTGTCTATCTGA	1254~1314
Atg3	NM_022488	75	AGGTATTACAGGAATAACGGAAGC	TCCTGTTATATTCCGAAGTTCTAAC	452~526
Atg4A	NM_052936	90	ACTGGCCTCCCTTTGTACCT	GACTCGTTGACCTCCTCAAAC	1196~1285
Atg4B	NM_013325	89	GTGCCAGCAAGTCAAAAAGC	GAAGTGTAGACCGGACGG	1099~1187
Atg4C	NM_032852	77	GCATAAAGGATTTCCCTCTTGA	GCTTTTTACCTAGGGTCG	1277~1353
Atg4D	NM_032885	85	ACGTTTCTCAGGACTGCACA	TGTCGGCTCACCTTCAGACA	935~1019
Atg5	NM_004849	107	CAACTTGTTTCACGCTATATCAGG	ACTGCAACCATTGACTGTTTCAC	403~509
Atg6	NM_003766	60	GGATGGTGTCTCTCGCAGAT	TACAGGTGTCTTTCACGGTT	387~446
Atg7	NM_006395	61	TGGCTGCTACTTCTGCAATG	TTGGTCTCTGGCCTGGAAC	1761~1821
Atg8	NM_007285	127	CCGTCGTTGTTGTTGTGCT	CCTTGTGTCTACGCACCTC	61~187
Atg8L	NM_031412	128	TGGGCCAACTGTATGAGGA	GTGGACCTGAACCCCCATC	512~639
Atg9A	NM_001077198	67	TGCATGCCCTCTATATGCAC	TTGGACTCGCCGTACATAC	2309~2393
Atg9B	NM_173681	85	GCACTGACTGAGACGACTCCT	TAGTGCTCGGACTCTTGACG	2841~2923
Atg10	NM_031482	74	GTCACATCTAGGAGCATCTACCC	AAAGCTCGATGGGAACCTAC	426~499
Atg12	NM_004707	87	TCTTCCGCTGCAGTTTCC	ACGATTTCCGACACCCTCTG	356~442
Atg16L1	NM_030803	96	TCGTTTCTGGGACATTCGAT	TTTGGGTCTTTCCTGACTCGA	1670~1765
Atg16L2	NM_033388	64	GGCCACAATGACCAGAAGAT	TGACGTGGGTCCAGTAGG	1475~1538
Atg18	NM_017983	62	GACCGTAGCCAGACCAAGTG	CCAATAAGACTCCTGCCGC	1240~1301
Atg21	NM_015610	71	CCGTGCACATCTTCAAACTC	TGGTGGACCTGGCCCATG	1028~1098
Prb1	NM_005039	85	GGCAGACCTTCCAGACCTC	CCTTCACTTATTCTTCTACTCTCACT	1007~1091
Vps15	NM_014602	69	CATCACTTCCTTTGCTGTGG	TGTTCGTCACCATGGTACCG	4010~4078
Vps34	NM_002647	67	GCAGTGGTGGAACAGATTCA	TTTGACCCTAACTGGGTAGT	2675~2741
